# HIV-1 induction of tolerogenic dendritic cells is mediated by cellular interaction with suppressive T cells

**DOI:** 10.3389/fimmu.2022.790276

**Published:** 2022-08-10

**Authors:** Cecilia Svanberg, Sofia Nyström, Melissa Govender, Pradyot Bhattacharya, Karlhans F. Che, Rada Ellegård, Esaki M. Shankar, Marie Larsson

**Affiliations:** ^1^ Molecular Medicine and Virology, Department of Biomedical and Clinical Sciences, Linköping University, Linköping, Sweden; ^2^ Department of Clinical Immunology and Transfusion Medicine, and Department of Biomedical and Clinical Sciences, Linköping University, Linköping, Sweden; ^3^ Unit for Lung and Airway Research, Institute of Environmental Medicine, Karolinska Institute, Stockholm, Sweden; ^4^ Division of Clinical Genetics, and Department of Biomedical and Clinical Sciences, Linköping University, Linköping, Sweden; ^5^ Infection Biology, Department of Life Sciences, Central University of Tamil Nadu, Thiruvarur, India

**Keywords:** dendritic cells, HIV-1, PDL1, cellular interactions, tolerogenic DCs, suppressor T cells

## Abstract

HIV-1 infection gives rise to a multi-layered immune impairment in most infected individuals. The chronic presence of HIV-1 during the priming and activation of T cells by dendritic cells (DCs) promotes the expansion of suppressive T cells in a contact-dependent manner. The mechanism behind the T cell side of this HIV-induced impairment is well studied, whereas little is known about the reverse effects exerted on the DCs. Herein we assessed the phenotype and transcriptome profile of mature DCs that have been in contact with suppressive T cells. The HIV exposed DCs from cocultures between DCs and T cells resulted in a more tolerogenic phenotype with increased expression of e.g., PDL1, Gal-9, HVEM, and B7H3, mediated by interaction with T cells. Transcriptomic analysis of the DCs separated from the DC-T cell coculture revealed a type I IFN response profile as well as an activation of pathways involved in T cell exhaustion. Taken together, our data indicate that the prolonged and strong type I IFN signaling in DCs, induced by the presence of HIV during DC-T cell cross talk, could play an important role in the induction of tolerogenic DCs and suppressed immune responses seen in HIV-1 infected individuals.

## Introduction

Human immunodeficiency virus (HIV) is primarily transmitted through heterosexual intercourse and causes immunological dysregulation in most infected individuals (1–3). This virus preferentially infects CD4^+^ tissue resident memory T cells located in the mucosa (4). In addition to CD4^+^T cells, HIV also targets dendritic cells (DCs). Even if these cells rarely become productively infected, they sense and are influenced by viral presence (5, 6). The DCs in the tissues provide the first line of defense against many microbes, including HIV-1 (7, 8), and their ablation has been linked to development of autoimmunity (9). Most HIV infected individuals have a good HIV specific T cell response that can keep the virus under control in the beginning of the disease. This response declines as HIV specific CD4^+^ T cells are infected and killed, and a general impairment of effector T cell function occurs (10–12). The interaction between DCs and other immune cells and the network of signaling cascades is complex (13–15). It will be the sum of the signaling by co-stimulatory and co-inhibitory molecules *via* interdependent routes, resulting from the microenvironment created by DC-specific stimulation and accessory cell interactions, that culminates in differentiation of effector or regulatory T cell responses (13, 16–18).

The extended response to prolonged antigenic stimulation such as chronic viral infection can lead to exhausted T cells with lost effector functions or give rise to regulatory/suppressive T cells (19–21). Induced regulatory/suppressive T cells are implicated in the control of inflammatory diseases (22–25). The subtypes of inducible regulatory T cells are complex and consist of many different subgroups (26).

The properties of the regulatory/suppressive cells activated depend on the microenvironment in which they were created/activated and signals they received, for instance, while prolonged IL-10 exposure can generate one subtype, exposure to transforming growth factor-β (TGF-β) can result in another (27–30). Regulatory/suppressive T cells can express several co-inhibitory molecules such as programmed cell death 1 (PD1), cytotoxic T lymphocyte antigen 4 (CTLA-4), lymphocyte activation gene 3 (LAG-3), T cell immunoglobulin and mucin domain-containing protein 3 (TIM3), CD160 and, B lymphocyte-induced maturation protein-1 (BLIMP-1), and depending on the frequency of expression of these markers, the T cells exhibit different levels of suppression (31, 32).

The presence of impaired/regulatory or tolerogenic DCs has been described in different settings such as tumor microenvironment and chronic infections (33, 34). Tolerogenic DCs can suppress T cell responses and induce differentiation of regulatory/suppressive T cells (35). These DCs can have elevated levels of IL-10, TGF-β, indoleamine 2,3 dioxygenase (IDO), cyclooxygenase-2 (COX-2), retinoic acid (35), and expression of co-inhibitory molecules, notably programmed death-ligand (PD-L) 1/PD-L2 (36), B7H3/B7H4 (37), HVEM (38), and/or galectin-9 (Gal-9) (39). It has been difficult to define a specific suppressive DC phenotype and correlate it to the level of T cell inhibition, which demonstrates the plasticity of DCs, i.e., ability to adapt to environmental cues. It has been indicated that during chronic conditions such as viral persistence, the type I interferons (IFNs) can have immunosuppressive effects and program individual immune cells such as DCs into cells with more immunosuppressive functions (40). Furthermore, direct exposure to HIV gp120 could impair the functional attributes of DCs (8, 41–44).

DC-signaling *via* co-stimulatory molecules, such as CD80, CD86, and CD40, or co-inhibitory molecules such as PDL1 during DC-T cell interactions shapes the T cell response. This engenders an enhanced bidirectional cell survival, by triggering T cell activation, which in turn activates the DCs to secrete cytokines such as IL-12 that polarizes the immune response towards TH1 (45–48). We have previously reported the effect HIV exerts on the DC-T cell crosstalk during priming and activation using an *in vitro* model and have shown that HIV-exposed mature DCs induce T cells with immune suppressive properties (49–51). In those studies, the T cells had increased expression of several co-inhibitory molecules, including PD1, CTLA4 and LAG-3 and CD160 (49–51).

In this study, we have explored the DC side in the DC-T cell coculture exposed to HIV to examine the effects exerted on the DCs by the suppressive T cells that develop in this system/setting. To our knowledge, there are no reports on how suppressive T cells affect DCs in the context of HIV. To this end, we set out to characterize the DC phenotype in the DC-T cell coculture with and without HIV by qPCR and flow cytometry. Furthermore, to identify what signaling pathways were associated with the HIV induced tolerogenic DC phenotype we performed transcriptomic analysis of the DCs separated from HIV exposed or unexposed DC-T cell cocultures. We found that DC interaction with T cells in the presence of HIV induced DCs with tolerogenic properties characterized by increased expression of co-inhibitory molecules such as PDL1, GAL-9, and herpes virus entry mediator (HVEM). This was not observed in DCs exposed to HIV without the T cell contact. At the transcriptome level we found a sustained type I IFN response and downstream type I IFN induced pathways in HIV-exposed DCs from DC-T cell cocultures, which may explain the induction of tolerogenic properties of the DCs after T cell interaction.

## Materials and methods

### Virus

HIV-1_BaL_/SUPT1-CCR5 CL.30 was produced using chronically infected cultures of ACVP/BCP Cell line (No. 204), originally derived by infecting SUPT1-CCR5 CL.30 cells (graciously provided by Dr J. Hoxie, University of Pennsylvania) with an infectious stock of HIV-1_BaL_ (NIH AIDS Research and Reference Reagent Program, Cat. No. 416, Lot 59155). Virus was purified by continuous flow centrifugation using a Beckman CF32Ti rotor at 30,000 rpm (~90000xg) at a flow rate of 6 liters/hour followed by banding for 30 minutes after sample loading. Sucrose density-gradient fractions were collected, virus-containing fractions pooled and diluted to <20% sucrose, and virus pelleted at ~100,000xg for 1 hour. The virus pellet was resuspended at a concentration of 1,000x relative to the cell culture filtrate and aliquots frozen in liquid N_2_ vapor.

### Reagents

RPMI1640 was supplemented with 10mM HEPES, 20µg/mL gentamicin (Fisher Scientific, Leicestershire, UK), 2mM L-glutamine (Sigma-Aldrich, St. Louis, MO, USA), and 1% plasma or 5% heat-inactivated pooled human serum (5% PHS, Innovative research, Novi, MI, USA). 100IU/mL recombinant human granulocyte-macrophage-colony stimulating factor (rhGM-CSF) and 300U/mL recombinant human interleukin-4 (rhIL-4) from Preprotech, London, UK, were used for the *in vitro* differentiation of DCs.

### Propagation and maturation of monocyte derived dendritic cells

Buffy coats or whole blood were obtained from healthy individuals (N=48) (ethical permit M173-07) and peripheral blood mononuclear cells (PBMCs) were isolated by density-gradient centrifugation over Ficoll-Paque™ (Amersham Pharmacia, Piscataway, NJ, USA). Monocytes were selected by plastic adherence after incubation of PBMCs in tissue culture dishes (BD Falcon, Franklin Lakes, NJ, USA) for one hour. The plates were then washed with RPMI1640 to remove non-adherent cells. The remaining adherent cells were cultured in 1% single donor plasma medium supplemented with rhGM-CSF (100 U/mL) and rhIL-4 (300 U/mL) and incubated in a 5% CO_2_ environment at 37°C. The rhGM-CSF and rhIL-4 cytokine supplement was replenished every second day to facilitate differentiation of CD14+ progenitor cells into immature DCs, and the cells were harvested on day 5. The purity and readiness of immature DCs was assessed by flow cytometry on a FACSCanto II (BD Immunocytometry Systems, San Jose, CA, USA). The DCs were phenotyped using phycoerythrin-conjugated (PE) monoclonal antibodies (mAbs) against CD83 and CD14 setting the cut-off for positive cells with the isotype control IgG_2a_ (BD Biosciences, Franklin Lakes, NJ, USA). Immature DCs were transferred to new plates at a concentration of 4x10^5^cells/mL, and maturation was induced by adding 30ng/ml polyinosinic acid: polycytidylic acid (Poly I:C) (Sigma-Aldrich, St. Louis, MO, USA). The maturation of DCs was assessed, after 24h of incubation, by CD83 surface expression by flow cytometry as described above.

### HIV exposure of mature DCs

Following the maturation of the DCs, 750 ng/mL p24 equivalents/mL corresponding to ~2 MOI of HIV-1_BaL_ (Lot No P4143, 4235, 4238, 4213), was added to the cells, and the DCs were incubated for an additional 24 hours. The unbound viruses were washed off the DCs by rinsing the plates with RPMI and changing the media before use in the assays. HIV unexposed mock mature DCs served as controls, and DC viability following HIV exposure was examined by trypan blue exclusion method (**Supplementary Figure 1**).

### Setup of the DC-T cell coculture

Cocultures of HIV-exposed and or unexposed mature DCs and naïve T cells were set in 96-well flat-bottom cell culture plates. The naïve T cells were isolated from allogenic non-adherent cells by a negative selection using magnetic beads coupled with anti-CD56, anti-CD19, anti-CD45RO and anti-CD14 magnetically tagged antibodies (MACS, Miltenyi Biotec, Auburn, CA) to deplete NK cells, B cells, memory T cells, and monocytes, respectively. The naïve T cell preparation was cocultured with mock or HIV-pulsed DCs at a 1:10 ratio and incubated at 37°C in a 5% CO_2_ incubator. Aliquots of the mature HIV-exposed and or unexposed mature DCs were stored frozen at -80°C in FBS (Gibco) containing 8% DMSO (Sigma Aldrich) and used for restimulation. The viability of the DCs after thawing was above 95%. The cocultures were restimulated on day 7 with the mock or HIV treated DCs from the same donor to reactivate the T cells. On day 8, the cocultures were harvested.

### T cell proliferation assay

After restimulation, the cocultures were pulsed with 2µCi/µl of [^3^H] labelled thymidine (PerkinElmer, Waltham, MA, USA) and incubated for about 16h before harvesting. The radioactive thymidine incorporated into the DNA of proliferating T cells was determined by liquid scintillation counting using a micro-β counter (PerkinElmer).

### Immunostaining of DCs and T cells

Directly conjugated mAbs against CD1c-BV421 (BD Biosciences), HLA-DR-PERCP (Invitrogen, Waltham, MA, USA), B7H3(CD276)-APC (Miltenyi, Stockholm, Sweden), B7H1-PE (Invitrogen), NOS1-FITC (Santa Cruz Biotechnology, Dallas, TX, USA), PDL2-APC (R&Dsystems), HVEM-PE (Invitrogen), Arginase 1-PerCP-eFluor 710 (Thermo fisher, Waltham, MA, USA), IDO-FITC (R&Dsystems), CD80-APC (Miltenyi), CD86-PE (BD Biosciences), CD85d (ILT4)-PerCP-eFluor 710 (Thermo fisher), COX2-FITC (Invitrogen), CD30L/TNFSF8-APC (R&Dsystems), Galectin-9-PE (Miltenyi), and B7-H4-Alexa Fluor 700 (R&Dsystems) were used for DC and T cell phenotyping before and after the DC–T cell interaction in the coculture. In brief the cells in the coculture were harvested, washed, and stained with the mAbs for 20-30 min at 4°C. Thereafter the cells were fixed with 4% PFA (Sigma Aldrich), permeabilized with 0.2% Saponin (Sigma Aldrich), and intracellular staining was performed at 4°C for 30-45 min. Data was acquired using FACS canto II, and the FlowJo software v9 (Treestar, OR, USA) was used for data analysis.

### Separation of DCs from the DC-T cell coculture and RNA sequencing

We harvested 10-15 million cells from the DC- T cell coculture day 8 and used two kinds of magnetically labelled beads to separate DCs from the T cells. Beads for removal of dead cells (Miltenyi) and beads targeting CD1c+ cells (Miltenyi) for positive selection of DCs and consequently negative selection of the T cells. All extracted cells using the magnetic separation was lysed for RNA purification and there was an excess of RNA from all the experiments used for RNA sequencing. Thereafter, a standardized number of DCs were lysed and RNA was extracted using Isolate II RNA Mini or Micro Kit (Bioline, London, UK). An amount of 5 ng of total RNA per sample was used for transcriptome amplification using NuGEN’s Ovation RNA-Seq V2 kit (San Carlos, CA, USA) according to the instruction from the manufacturer. Library quality and size distribution was determined using Agilent Bioanalyzer 2100. A total of 16 libraries from 8 different donors (two conditions/donor) were prepared and ran in two different runs on the Illumina NextSeq500 platform (San Diego, CA, USA). FASTQ files were analyzed using UPPMAX, and quality was checked using the fastQC and multiQC programs. After quality controls libraries were trimmed using trimmomatic followed by mapping of the libraries using STAR to the human reference genome hg38. FeatureCounts was applied for retrieving counts for each mapped gene. Using R/DeSeq2 data was normalized and differentially expressed genes were determined. Thereafter, the data was visualized using Ingenuity Pathway Analysis (IPA, Qiagen), R analysis and Gene Ontology (GO) Enrichment Analysis (Geneontology.org). A p-value cut-off of 0.05 was set as significant for affected molecules/pathways.

### CFSE staining of naïve T cells and assessment of T cell priming by tolerogenic dendritic cells

MACS enriched naïve T cells in a concentration of 5x10^7^ cells/mL were stained with 10µM CFSE (Sigma Aldrich) in 0.2% FBS PBS at 37°C for 10 min before washing twice with RPMI containing 10%FCS. Cells were thereafter stimulated with DCs separated from day 8 DC- T cell cocultures after 24h culturing (see method for DC separation above) in a ratio of 1:5 DC:T cells. CFSE dilution and PDL1 protein expression was measured with flow cytometry after 7 days of culture.

### Quantitative polymerase chain reaction

Cryopreserved cell lysates of DCs (24h exposure) enriched from DC- T cell cocultures or whole cocultures were used for RNA extraction, cDNA synthesis and quantitative (q) PCR analysis. RNA was extracted using Isolate II RNA Mini or Micro Kit (Bioline, UK) and the and first strand cDNA was synthesized using the superscript III RT kit (Invitrogen^™^, USA), both according to the instructions from the manufacturers. The primers used for qPCR were retrieved from the primer bank found at http://pga.mgh.harvard.edu/primerbank/ (See **Supplementary Table 1** for genes and primers). The samples in the PCR plate were run in duplicates. Expression of negative co-stimulatory molecules together with β-actin and GAPDH endogenous controls were analyzed using CFX96 Touch Real-Time system (BIO-RAD Inc.) in 96-well MicroAmp^®^ optical fast-plates (Applied Biosystems^®^) and Fast SYBR Green^®^ Master Mix (Applied Biosystems^®^). Results were firstly normalized according to the 2^-ΔΔCt^ method, where *Ct* refers to the threshold value, and thereafter further normalized as ratios with the total sum of both conditions for each donor set to 100%.

### Statistical analysis

All data were analyzed using MS Excel and GraphPad Prism 9 software (GraphPad, La Jolla, CA, USA). The qPCR data was normalized, and statistical significance determined by unpaired t test. For data normalized with the control (unexposed DCs) value set to one was Wilcoxon test used to determine statistical significance between groups. For all tests, a *P* value of <0.05 was considered significant, and the measure of significance was represented by *P<0.05, **P<0.005 and ***P<0.001.

## Results

### Presence of HIV during DC priming of naïve T cells leads to decreased T cell proliferation

**Figure 1:**
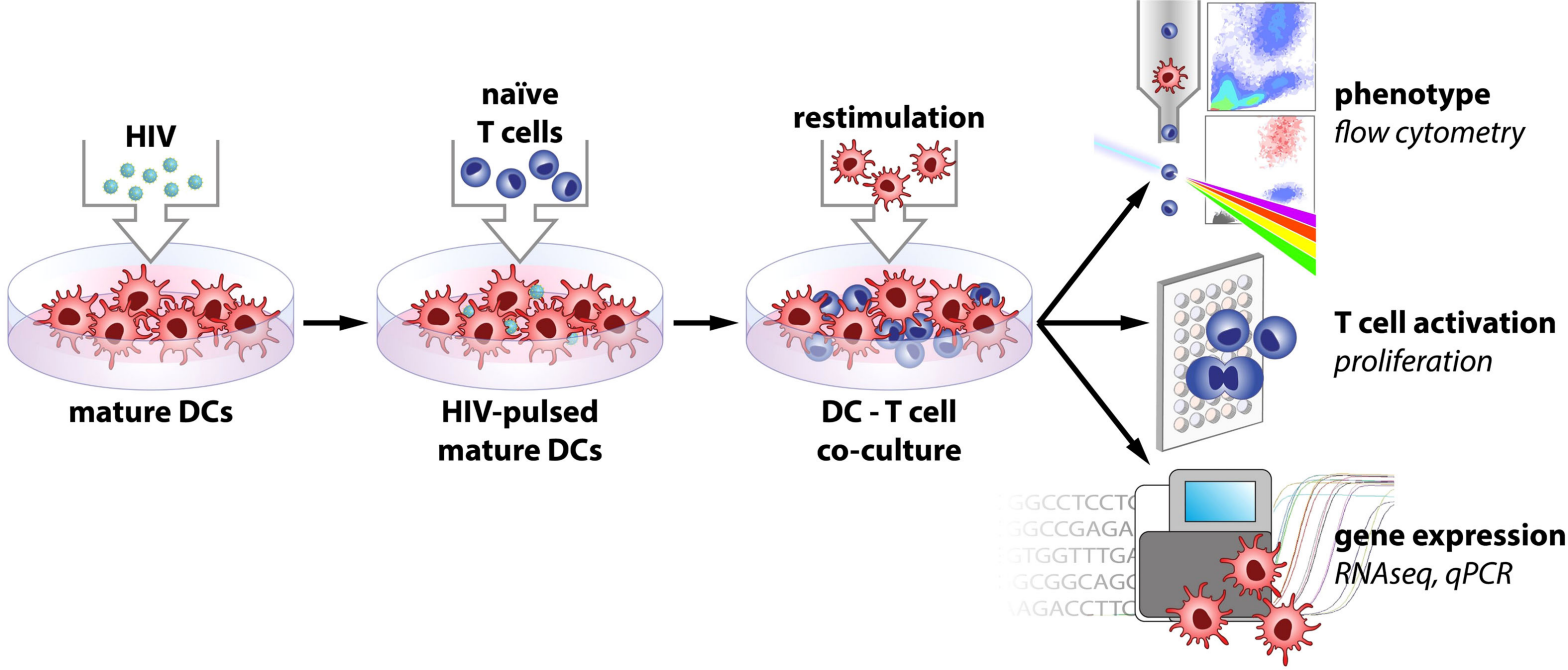
Model of experimental setup.

**Figure 2:**
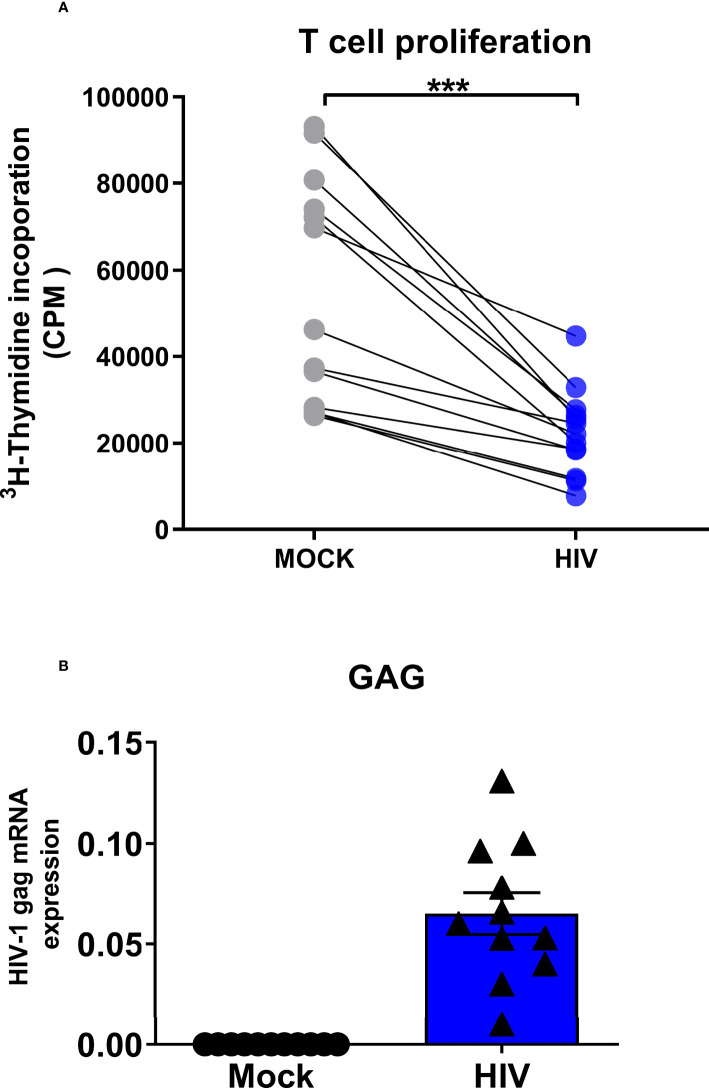
Presence of infectious HIV impairs the ability of DCs to prime naïve T cell responses. Mature DCs were pulsed with infectious HIV-1_BaL_ (CCR5-tropic) overnight, washed twice, and cocultured with naïve bulk T cells at a ratio of 1:10 (10^4^ DCs and 10^5^ T cells). The primed cultures were restimulated with 10000 DCs per well after 7 days of coculture, and T cell proliferation was determined by [^3^H] thymidine incorporation into the DNA of proliferating T cells day 8 by liquid scintillation counting using a micro-β counter. Values expressed as counts per minute (CPM) **(A)**. The levels HIV gag transcripts at day 8 in the HIV exposed and unexposed DC-T cell cocultures were measured by PCR **(B)**. N=11-13. ^***^ *P*<0.001, paired t-test.

In an HIV infected individual there will be virions in the lymphoid tissue, affecting the priming and reactivation of T cells as well as the quality of the immune responses (3, 52–54). Here we tried to mimic the *in vivo* setting in the lymph nodes by using an experimental allogenic HIV priming model with mature DCs and naïve T cells (**Figure 1**). In this setting we assessed the effects that DC-T cell crosstalk exerts on the DCs’ phenotype and transcriptomic profile. Unexposed or HIV exposed mature DCs were cocultured with CD3^+^CD45RA^+^ bulk naïve T cells. On day 7, cocultures were restimulated with the same donor-DCs added on day 1, and T cell proliferation was measured by [^3^H] thymidine incorporation. HIV exposure of mature DCs resulted in impaired T cell priming compared to the mock treated mature DCs, illustrated by decreased proliferation (**Figure 2A**). DCs exposed to HIV before the coculture with T cells had the same viability and expression of CD83 as the untreated cells (**Supplementary Figure 1**). There was a low level of HIV gag transcripts in cocultures of HIV exposed DCs (**Figure 2B**), confirming our previous data of a minimal level of HIV replication in the system (49).

### HIV exposure of mature DCs give rise to suppressive DCs following cellular crosstalk with T cells

**Figure 3:**
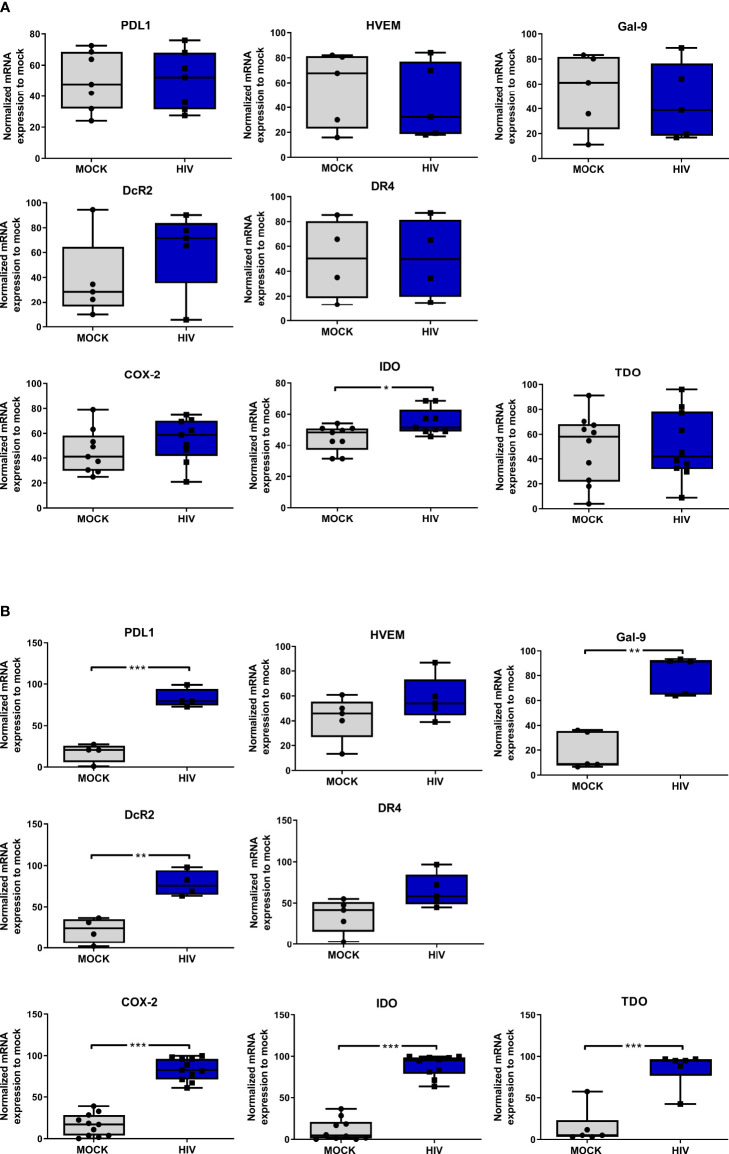
Presence of HIV in DC-T cells coculture give rise to DCs with an increased gene expression of factors associated with a tolerogenic phenotype. Mature DCs were pulsed with mock and HIV-1_BaL_ (HIV), and cultured 24h, washed and studied for gene expression levels of **(A)** PDL1, HVEM, Gal-9, DcR2, DR4, COX-2, IDO and TDO by DCs. N=7. DC-T cell cocultures, with or without HIV were harvested after 8 days. The gene expression levels in the day 8 coculture of **(B)** PDL1, HVEM, Gal-9, DcR2, DR4, COX-2, IDO, and TDO examined by PCR (N=5-11). The expression was normalized as ratios of 100%. Shown are min to max and all data points. ^*^ *P*<0.05, ^**^ *P*<0.005, ^***^ *P*<0.001, unpaired t-test.

**Figure 4:**
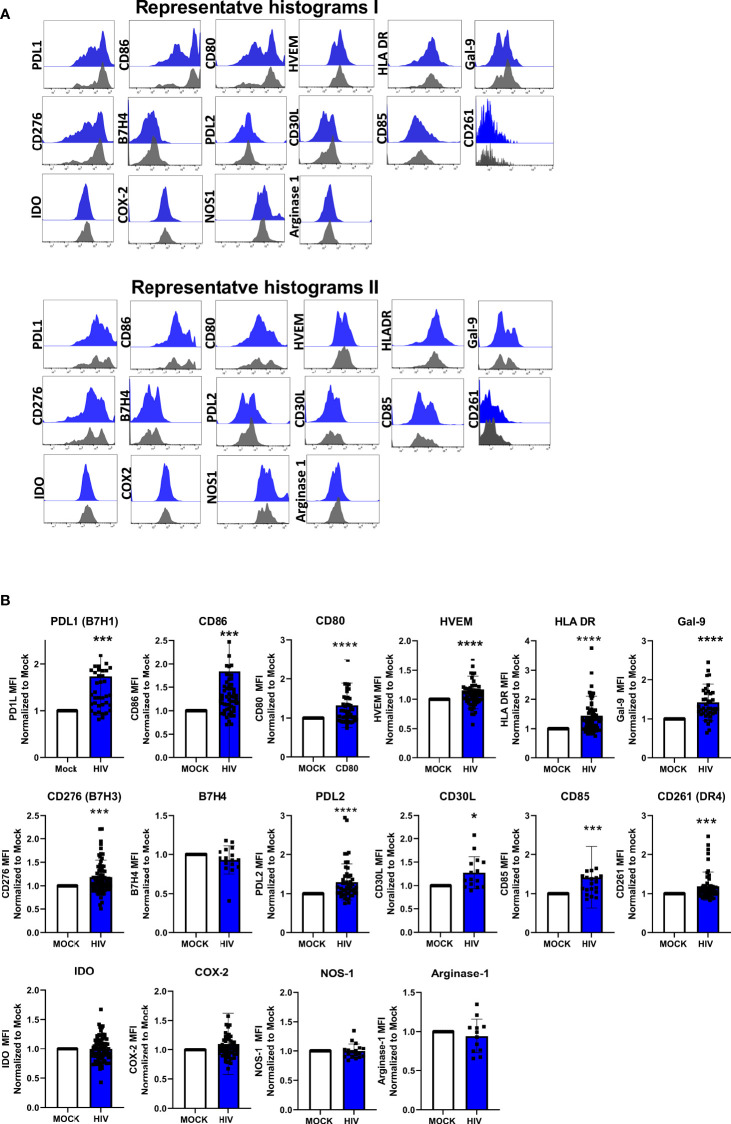
Presence of HIV in DC-T cells coculture give rise to DCs with upregulated protein expression of molecules associated with a tolerogenic phenotype. DC-T cell cocultures, unexposed or exposed to HIV were harvested after 8 days. DCs in the coculture were identified by expression of CD1c. DC expression-levels of PDL1, CD86, CD80, HVEM, HLA DR, Gal-9, CD276/B7H3, B7H4, PDL2, CD30L, CD85, CD261/DR4, IDO, COX-2, NOS1, and arginase 1. Representative histograms of 2 donors show the expression of these markers on CD1c positive DCs **(A)**. Graphs of normalized MFI values of the markers on CD1c positive DCs **(B)**. The experiments (N=12-60) were normalized by setting each donor’s mock value to one and compared to the corresponding HIV value, and Wilcoxon test performed. Shown are min to max and all data points. ^*^ *P*<0.05, ^***^ *P*<0.001, ^****^ *P*<0.0001.

It has been shown that DC phenotype and function can be further modified by environmental stimuli and cellular interactions (6, 55–57). We used qPCR to investigate the effect that cellular interaction with T cells had on mature DCs exposed to HIV, with focus on established co-inhibitory factors known to be expressed by DCs with a tolerogenic phenotype. We exposed matured DCs to HIV-1_BaL_ or left them unexposed for 24h and found that the HIV exposure alone, had no effect on DC gene expression levels of PDL1, decoy receptor (DcR) 2, death receptor 4 (DR4), Gal-9, HVEM, tryptophan 2,3-dioxygenase (TDO), or cyclooxygenase (COX)-2 (**Figure 3A**). The only factor that increased in HIV exposed DCs compared to unexposed (mock) DCs was IDO (**Figure 3A**). We further investigated the expression of co-inhibitory factors and enzymes, in the DC-T cell cocultures of HIV exposed or unexposed DCs after restimulation. We found increased gene expression levels of Gal-9, PDL1, and DcR2 (**Figure 3B**), as well as COX-2, IDO, and TDO in restimulated cocultures with DCs exposed to HIV (**Figure 3B**). PDL1 and IDO levels were already elevated at 5h after restimulation of the coculture with HIV exposed DCs (**Supplementary Figure 2**). We next investigated if the mRNA expression findings were reflected at the protein level by performing flow cytometry analysis of the DCs in the DC-T cell cocultures after 24h of restimulation (**Figures 4A, B**). We found significantly increased levels of PDL1, CD86, CD80, HVEM, HLA DR, Gal-9, B7H3 (CD276), PDL2, CD85, CD30L, HLA-DR, CD80 and CD86 and DR4 in HIV exposed DCs compared to unexposed DCs in restimulated cocultures (**Figures 4A, B**). HIV exposure did not significantly alter the protein expression of B7H4, IDO, COX-2, NOS1, and arginase 1 (**Figures 4A, B**).

### Dendritic cells separated from HIV exposed DC-T cell coculture suppress priming of new naïve T cells

**Figure 5:**
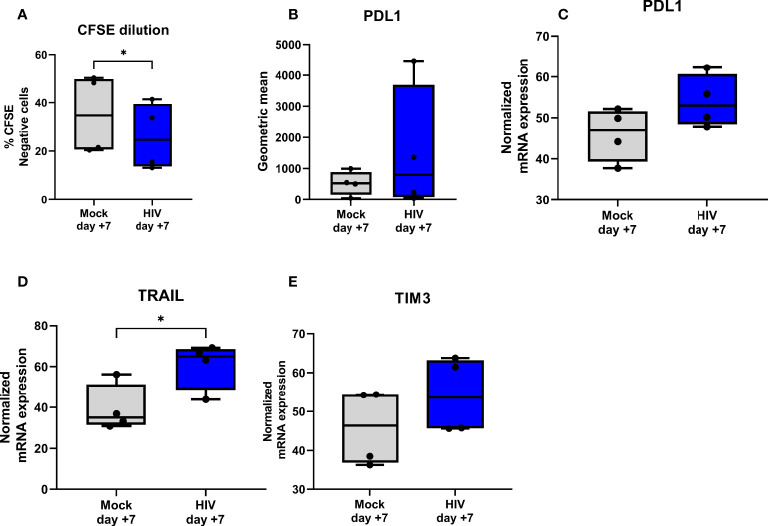
DCs separated from HIV exposed DC-T cell coculture can suppress priming of naïve T cells and induce expression of molecules associated with a tolerogenic DC and T cell phenotype. Mock or HIV exposed DCs was separated from DC-T cell cocultures after 24h culture and cultured with naïve T cells at 1:5 ratio (N= 4-6). After 7 days of culture proliferation was measured by CFSE dilution **(A)** and phenotype investigated on protein level by flow cytometry for PDL1 **(B)** and on gene level by qPCR for PDL1 **(C)**, TIM3 **(D)** and TRAIL **(E)**. The qPCR data was normalized as ratios of 100%. Shown are min to max and all data points. ^*^ *P*<0.05, unpaired t-test.

DCs conditioned in the day 8 HIV DC-T cell coculture for 24h induced less proliferation (day 7) of naïve T cells compared to unexposed DC-T cell derived DCs (**Figure 5A**). In addition, the levels of PDL1, both on mRNA and protein level, was assessed at day 7 but we found no significant differences even if the HIV DC-T cell coculture had a tendency towards higher expression (**Figures 5B, C**). TRAIL, a known co-inhibitory molecule on T cells was significantly higher in the HIV exposed coculture (**Figure 5D**) and TIM3 had a tendency to be increased in the HIV exposed coculture, but this was not significant (**Figure 5E**).

### The transcriptomic profile showed type I IFN pathway activation in the DCs from HIV conditioned DC-T cell cocultures

**Figure 6:**
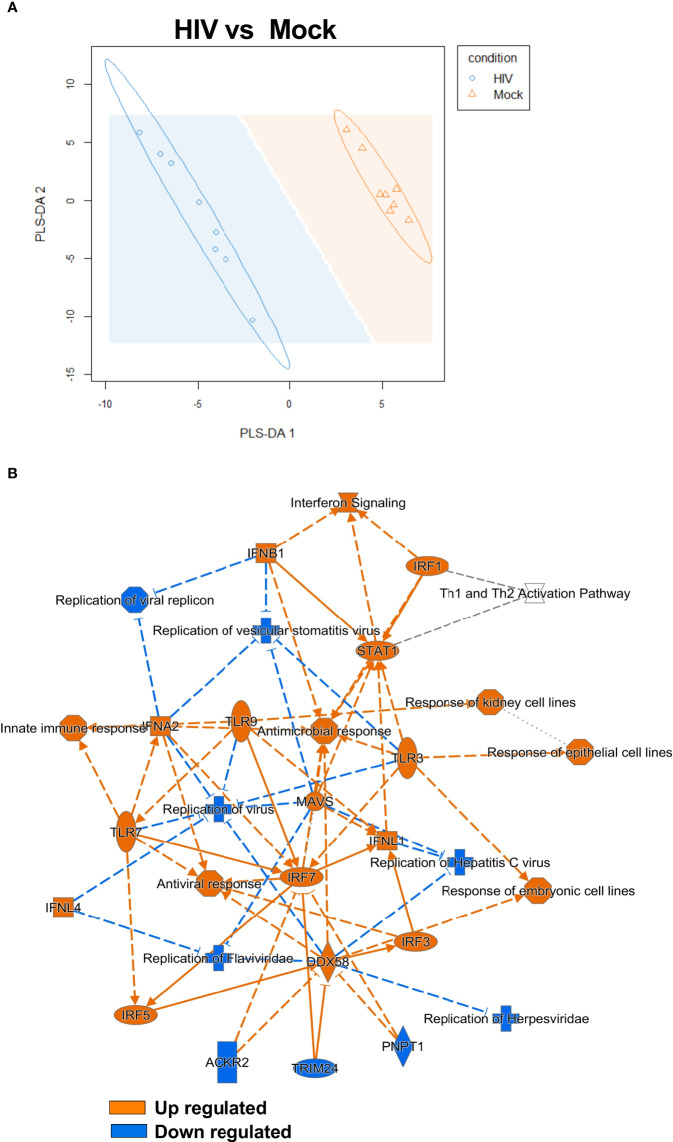
Transcriptomic data revealed a clear type I IFN signaling profile in HIV exposed DC cocultures. PLS-DA analysis was performed to model relationship between HIV exposed and unexposed HIV DCs after coculture with T cells (N=8) **(A)**. Transcriptomic data set including 8 individual donors/experiments were analyzed using Ingenuity Pathway Analysis (IPA). IPA selected pathways were visualized as network with a threshold for p-values set to -log 1.3 (p<0.05) and presented as positive activation Z-score in orange and negative in blue **(B)**.

After finding elevated protein levels of an array of co-inhibitory factors on DCs from DC-T cell coculture exposed to HIV and confirming that these cells had suppressive properties (**Figure 5**), we wanted to elucidate signaling pathways involved in this altered phenotype. To do this we assessed the transcriptomic profile of the DCs in the DC-T cell coculture by sorting the DCs after 24h restimulation and perform RNA sequencing (see model **Figure 1A**). Partial least squares discriminant analysis (PLS-DA) model was used to explore if there were underlying relationships between the DCs from HIV exposed and unexposed DC-T cell cocultures. The PLS-DA revealed a clear separation between the DCs from the untreated mock compared to the HIV exposed DC-T cell coculture (**Figure 6A**). To further examine the transcriptomic data, we used IPA to analyze the genes and pathways that separated the DCs sorted from the HIV coculture vs mock coculture. The graphic summary/overview of the main pathways affected clearly illustrated a strong activation of type I IFN response, and an upregulation of innate immune factors and pathways associated with inhibition of viral replication (**Figure 6B**). The Interferon signaling, T cell exhaustion signaling pathway, Role of PKR in Interferon Induction and Antiviral Response, Death Receptor Signaling, and Role of Pattern Recognition Receptors in Recognition of Bacteria and Viruses, were among the top pathways with positive Z-scores (**Table 1**). The canonical pathways that were inhibited/decreased included the ICOS-ICOSL Signaling in T helper cells, and TGF-β Signaling, PKCβ Signaling in T lymphocytes, Systemic Lupus Erythematosus In T Cell Signaling Pathway, and STAT3 Pathway (**Table 2**).

**Table 1:** Canonical pathways with positive Z-score.

Canonical Pathways	-log (p-value)	z-score	Molecules
**Interferon Signaling**	6.37	3	IFI35, IFI6, IFITM2, ISG15, JAK2, MX1, OAS1, STAT1, STAT2
**Retinoic acid Mediated Apoptosis Signaling**	3.63	2.828	PARP10, PARP11, PARP12, PARP14, PARP8, PARP9, TIPARP, TNFSF10
**Necroptosis Signaling Pathway**	1.92	2.53	CAPN2, CASP10, EIF2AK2, FASLG, PGAM5, RBCK1, STAT1, STAT2, TNFSF10, ZBP1
**Role of PKR in Interferon Induction and Antiviral Response**	2.26	2.333	DDX58, EIF2AK2, FASLG, IFIH1, IL24, MYD88, PDGFD, STAT1, STAT2
**Death Receptor Signaling**	5.06	2.309	CASP10, FASLG, HTRA2, LMNA, PARP10, PARP11, PARP12, PARP14, PARP8, PARP9, TIPARP, TNFSF10
**Role of Pattern Recognition Receptors in Recognition of Bacteria and Viruses**	4.6	2.121	CD40LG, CSF2, DDX58, EIF2AK2, FASLG, IFIH1, IL5, IRF7, MYD88, NOD2, OAS1, PRKCE, TLR7, TNFSF10, TNFSF13B
**Type I Diabetes Mellitus Signaling**	4.24	2	FASLG, GAD1, HLA-DOB, HLA-DQA1, HLA-DRA, HLA- DRB1, HLA-DRB5, HLA-E, JAK2, MYD88, PRF1, STAT1
**T Cell Exhaustion Signaling Pathway**	6.38	1.414	BTLA, HLA-DOB, HLA-DPA1, HLA-DPB1, HLA-DQA1, HLA-DQA2, HLA-DRA, HLA-DRB1, HLA-DRB5, HLA-E, IL12RB2, JAK2, LAG3, LGALS9, RALB, RRAS2, STAT1, STAT2, TGFBR3
**Systemic Lupus Erythematosus In B Cell Signaling Pathway**	5.57	1.877	CCND3, CD22, CD40LG, CSF2, FASLG, IFIH1, IL5, IRF7, ISG15, ISG20, JAK2, MYD88, PIK3AP1, PRKCE, RALB, RASGRP3, RRAS2, STAT1, STAT2, STING1, TLR7, TNFSF10, TNFSF13B
**Role of RIG1-like Receptors in Antiviral Innate Immunity**	2.9	1.633	CASP10, DDX58, DHX58, IFIH1, IRF7, TRIM25
**Natural Killer Cell Signaling**	3.4	1.604	CD48, COL1A1, FASLG, HLA-E, IL12RB2, IL18R1, IL18RAP, JAK2, KLRB1, KLRC1, MAP3K8, MYD88, RALB, RRAS2, TNFSF10
**Neuroinflammation Signaling Pathway**	1.69	1.069	FASLG, GAD1, HLA-DOB, HLA-DQA1, HLA-DRA, HLA- DRB1, HLA-DRB5, HLA-E, HMOX1, IRF7, JAK2, MYD88, STAT1, TGFBR3, TLR7
**Activation of IRF by Cytosolic Pattern Recognition Receptors**	4.26	1	ADAR, DDX58, DHX58, IFIH1, IRF7, ISG15, STAT1, STAT2, ZBP1
**PD-1, PD-L1 cancer immunotherapy pathway**	3.77	0.707	HLA-DOB, HLA-DPA1, HLA-DPB1, HLA-DQA1, HLA- DQA2, HLA-DRA, HLA-DRB1, HLA-DRB5, HLA-E, IL2RA, JAK2
**Apoptosis Signaling**	2.19	0.707	CAPN2, CASP10, FASLG, HTRA2, LMNA, PRKCE, RALB, RRAS2
**Prolactin Signaling**	1.61	0.447	JAK2, NMI, PRKCE, RALB, RRAS2, STAT1
**TREM1 Signaling**	2.33	0.378	CIITA, CSF2, JAK2, MYD88, NLRC5, NOD2, TLR7
**Salvage Pathways of Pyrimidine Ribonucleotides**	1.72	0.378	AK4, CDK6, CMPK2, EIF2AK2, MAP3K8, PRKCE, UCK2
**Crosstalk between Dendritic Cells and Natural Killer Cells**	4.47	0.302	CD40LG, CSF2 ,FASLG, HLA-DRA, HLA-DRB1, HLA- DRB5, HLA-E, IL15RA, PRF1, TLR7, TNFSF10
**MSP-RON Signaling In Macrophages Pathway**	6.28	0.258	CIITA, GAB2, HLA-DOB, HLA-DPA1, HLA-DPB1, HLA- DQA1, HLA-DQA2, HLA-DRA, HLA-DRB1, HLA-DRB5, ITGAM, JAK2, RALB, RRAS2, STAT1
**Tumor Microenvironment Pathway**	2.9	0.277	COL1A1, CSF1, CSF2, FASLG, FN1, HLA-E, IGF1, JAK2, LGALS9, MMP21, PDGFD, RALB, RRAS2

**Table 2:** Canonical pathways with negative Z-score.

Canonical Pathways	-log (p-value)	z-score	Molecules
**iCOS-iCOSL Signaling in T Helper Cells**	2.99	-2.333	CD4, CD40LG, GAB2, HLA-DOB, HLA-DQA1, HLA-DRA, HLA-DRB1, HLA-DRB5, IL2RA, PLEKHA1
**TGF-β Signaling**	1.3	-2.236	IRF7, RALB, RRAS2, RUNX2, SMAD9, VDR
**PKCθ Signaling in T Lymphocytes**	1.54	-1.667	CD4, HLA-DOB, HLA-DQA1, HLA-DRA, HLA-DRB1, HLA- DRB5, MAP3K8, RALB, RRAS2
**SPINK1 General Cancer Pathway**	1.92	-1.633	JAK2, MT1E, MT1F, MT2A, RALB, RRAS2
**Systemic Lupus Erythematosus In T Cell Signaling Pathway**	1.62	-1.5	CASP10, CASP4, CD40LG, ELF1, FASLG ,HLA-DOB, HLA- DPA1, HLA-DPB1, HLA-DQA1, HLA-DQA2, HLA-DRA, HLA-DRB1, HLA-DRB5, HLA-E, RALB, RRAS2
**Cardiac Hypertrophy Signaling (Enhanced)**	2.83	-1.279	ADCY1, ADCY2, CD40LG, CSF2, FASLG, IGF1, IL12RB2, IL15RA, IL18R1, IL18RAP, IL1R2, IL2RA, IL4R, IL5, JAK2, MAP3K8, PDE4A, PDE7B, PRKCE, RALB, RRAS2,RYR1, TGFBR3, TNFSF10, TNFSF13B, WNT5B
**Regulation of Cellular Mechanics by Calpain Protease**	1.78	-1	CAPN2, CCNA1, CDK6, RALB, RRAS2, VCL
**STAT3 Pathway**	3.43	-0.816	IGF1 ,IL12RB2, IL15RA, IL18R1, IL18RAP, IL1R2, IL2RA, IL4R, JAK2, RALB, RRAS2, TGFBR3
**Sphingosine-1-phosphate Signaling**	1.36	-0.816	ACER2, ADCY1, ADCY2, CASP10, CASP4, PDGFD, S1PR5
**Gαi Signaling**	1.23	-0.816	ADCY1, ADCY2, CCR4, GNG4, RALB, RRAS2, TBXA2R
**Coronavirus Pathogenesis Pathway**	1.24	-0.707	ADAM17, DDX58, FASLG, IRF7, STAT1, STAT2, STING1, TRIM25
**Calcium-induced T Lymphocyte Apoptosis**	3.34	-0.707	CAPN2, CD4, HLA-DOB, HLA-DQA1, HLA-DRA, HLA- DRB1, HLA-DRB5, PRKCE
**Coronavirus Pathogenesis Pathway**	1.24	-0.707	ADAM17, DDX58, FASLG, IRF7, STAT1, STAT2, STING1, TRIM25
**Dendritic Cell Maturation**	2.73	-0.577	CD40LG, COL1A1, CSF2, HLA-DOB, HLA-DQA1, HLA- DRA, HLA-DRB1, HLA-DRB5, HLA-E, JAK2, MYD88, STAT1, STAT2
**Macropinocytosis Signaling**	1.73	-0.447	CSF1, MET, PDGFD, PRKCE, RALB, RRAS2
**GM-CSF Signaling**	1.35	-0.447	CSF2, JAK2, RALB, RRAS2, STAT1
**IL-15 Signaling**	1.25	-0.447	CSF2, IL15RA, JAK2, RALB, RRAS2
**NF-κB Activation by Viruses**	1.23	-0.447	CD4, EIF2AK2, PRKCE, RALB, RRAS2
**PPARα/RXRα Activation**	1.4	-0.333	ADCY1, ADCY2, FASN, HELZ2, IL18RAP, IL1R2, JAK2, RALB, RRAS2, TGFBR3
**Th1 Pathway**	7.4	-0.302	CD4, CD40LG, CD8A ,HLA-DOB, HLA-DPA1, HLA-DPB1, HLA-DQA1, HLA-DQA2, HLA-DRA, HLA-DRB1, HLA- DRB5, IL12RB2, IL18R1, JAK2, KLRC1, LGALS9, STAT1

To follow up the strong IFN signaling profile seen in the RNA sequencing data we measured mRNA expression levels in the day 8 cocultures by qPCR for IFNα (**Supplementary Figure 3**) and the IFN inducible genes (ISGs) IFITM2, MX2, and IFI16 (**Supplementary Figure 3**) as well as MX2 at day 7 in the coculture primed with tolerogenic DCs separated after 24h from day 8 DC-T cell coculture and verified activation of the type I IFN pathway.

### Top canonical pathways revealed that interferons regulated signaling in DCs from HIV conditioned DC-T cell cocultures

**Figure 7:**
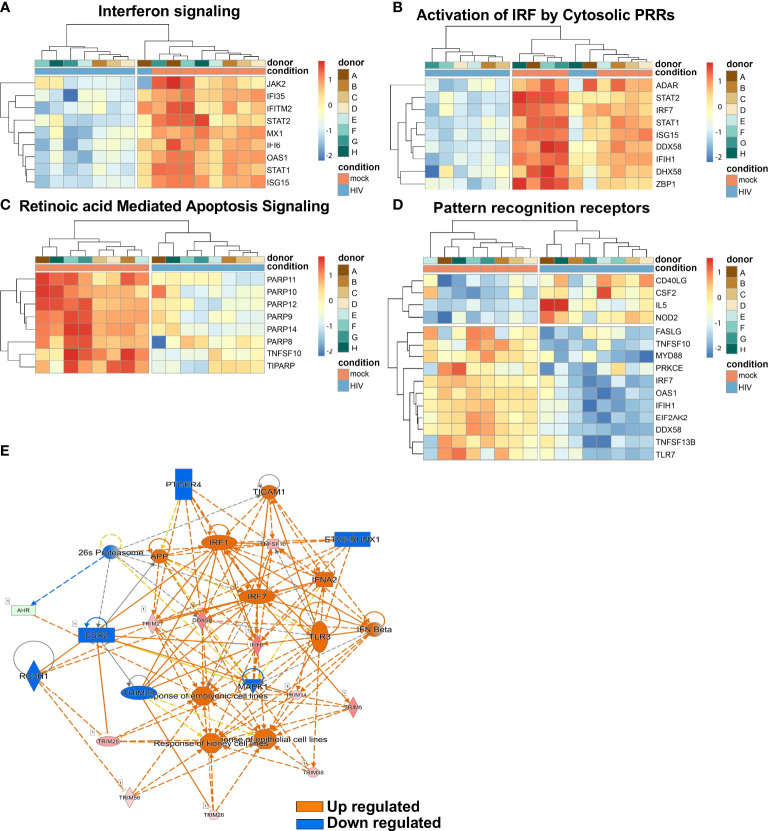
The top infection, inflammation, and immunology pathway regulators in HIV exposed DC cocultures. Transcriptomic data set of HIV exposed and unexposed DCs set including data from 8 individual donors/experiments was analyzed using pheatmap package to provide hierarchic clustered heatmaps of Interferon signaling **(A)**, Activation by Cytosolic PRRs **(B)**, Retinoic Acid Mediated Apoptosis Signaling **(C)** and Pattern recognition receptors **(D)** pathways with genes recognized in IPA to belong to these pathways. Transcriptomic data set from 8 individual donors/experiments was analyzed for Pathway regulators limited to infection, inflammation and immunology and were visualized as network with a threshold for p-values set to -log 1.3 (p<0.05) and presented as positive activation Z-score in orange and negative in blue **(E)**.

Several top pathways of DCs from the mock and HIV treated cocultures were next examined as transcriptome heatmap profiles displaying all donors. The Interferon signaling pathway showed upregulation of JAK2, IFI36 and IFITM (**Figure 7A**). In addition, the pathway Activation of IRF by Cytosolic PRRs showed upregulation of ADAR, STAT2 and IRF7 (**Figure 7B**). A clear difference in HIV exposed DCs versus untreated DCs was the activation of the Retinoic Acid Mediated Apoptosis Signaling pathway (**Figure 7C**) in which an increase in the signaling molecules PARP11, PARP10, and PARP12 were seen in the HIV exposed DC groups. The pathway Pattern recognition receptors showed increased expression of FASLG, TNFS10 and MYD88 (**Figure 7D**).

Next, we assessed top upstream regulators and factors predicted to be activated including IFNa2, IFNL1, IFNβ, IFNγ, and the transcriptional regulators IRF7, STAT1, and IRF1 (**Table 3**). Top upstream regulators predicted to be inhibited were MAPK1, PNPT1, and IL1RN and the transcriptional regulators NKX2-3, TRIM24, and ETV6-RUNX1 (**Table 4**). When performing an analysis focusing on inflammation and infection associated factors in IPA, we found a strong Z-score for IFNβ, IFNA2, IRF1, and IRF7 signaling (**Figure 7E**). Taken together these data suggest a strong activation of IFN signaling that might play a role in the induction of tolerogenic DCs and impaired T cell proliferation in the HIV DC-T cell coculture.

**Table 3:** Upstream regulators with Positive Z-score.

Upstream Regulator	Molecule Type	Activation z-score	p-value of overlap
**IFNA2**	cytokine	7.209	1.66E-49
**IRF7**	transcription regulator	6.668	5.52E-38
**IFNL1**	cytokine	6.355	7.77E-44
**Interferon alpha**	group	6.324	1.92E-63
**IFNG**	cytokine	5.936	7.82E-43
**PRL**	cytokine	5.926	2.16E-40
**IFNB1**	cytokine	5.548	1.25E-27
**STAT1**	transcription regulator	5.515	1.64E-31
**IRF3**	transcription regulator	5.403	2.05E-22
**poly rI:rC-RNA**	biologic drug	5.384	2.76E-28
**IRF1**	transcription regulator	5.002	1.48E-15
**Ifnar**	group	4.964	2.41E-22
**EIF2AK2**	kinase	4.647	3.44E-14
**IFN Beta**	group	4.496	2.33E-21
**IRF5**	transcription regulator	4.16	3.35E-12
**Ifn**	group	4.142	3.11E-12
**mir-96**	microRNA	4.101	4.09E-16
**mir-183**	microRNA	4.088	7.34E-17
**IL1B**	cytokine	4.041	1.78E-13
**RNY3**	other	4	1.11E-18
**TGM2**	enzyme	3.914	2.47E-16
**IFN type 1**	group	3.798	3.27E-13
**IFNA1/IFNA13**	cytokine	3.769	4.06E-11
**TICAM1**	other	3.645	5.93E-07
**SAMSN1**	other	3.606	1.44E-06
**MAVS**	other	3.36	7.84E-12
**DOCK8**	other	3.317	3.09E-06
**IFNA4**	cytokine	3.251	1.27E-07

**Table 4:** Upstream regulators with Negative Z-score.

Upstream Regulator	Molecule Type	Activationz-score	p-value of overlap
**NKX2-3**	transcription regulator	-5.94	4.65E-31
**MAPK1**	kinase	-5.802	1.51E-35
**TRIM24**	transcription regulator	-5.696	1.01E-24
**PNPT1**	enzyme	-4.666	1.92E-21
**IL1RN**	cytokine	-4.52	3.12E-18
**RC3H1**	enzyme	-4.146	1.31E-18
**STAT6**	transcription regulator	-4.104	4.01E-13
**ETV6-RUNX1**	fusion gene/product	-4.065	2.1E-13
**ACKR2**	G-protein coupled receptor	-4	1.49E-14
**miR-182-5p (and miRNAs w/seed UUGGCAA)**	mature microRNA	-3.846	5.43E-19
**PTGER4**	G-protein coupled receptor	-3.554	5.60E-11
**ESR2**	ligand-dependent nuclear receptor	-3.496	5.05E-06
**PGR**	ligand-dependent nuclear receptor	-3.45	5.2E-144
**dexamethasone**	chemical drug	-3.015	7.55E-15
**IL4**	cytokine	-2.942	5.43E-20
**TREX1**	enzyme	-2.922	2.12E-09
**SOCS1**	other	-2.887	4.04E-10
**IRF4**	transcription regulator	-2.754	7.31E-14
**TCF4**	transcription regulator	-2.72	4.28E-12
**IL10RA**	transmembrane receptor	-2.692	3.13E-03
**IKZF3**	transcription regulator	-2.655	4.13E-06
**fontolizumab**	biologic drug	-2.646	1.95E-07
**TAB1**	enzyme	-2.646	2.91E-05
**AIRE**	transcription regulator	-2.646	7.48E-03
**lysophosphatidyl- choline**	chemical - other	-2.621	3.93E-3
**USP18**	peptidase	-2.592	1.88E-05
**SIRT1**	transcription regulator	-2.513	3.47E-11
**WNT3A**	cytokine	-2.443	7.02E-02

### DCs from HIV conditioned DC-T cell cocultures have altered T cell activating profile

**Figure 8:**
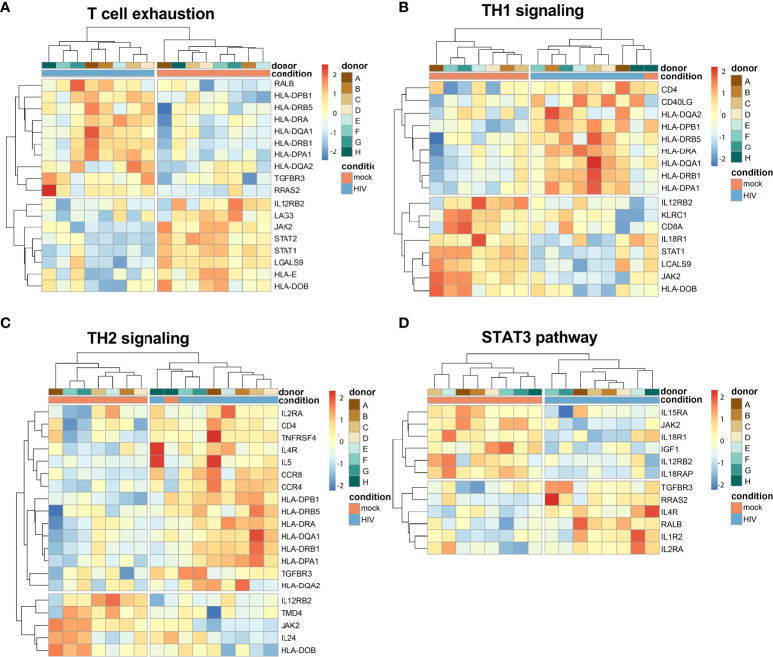
Transcriptomic data shows an activation of molecules involved in T cell exhaustion. Transcriptomic data set of HIV exposed and unexposed DCs with data from 8 individual donors/experiments was analyzed using heatmap package to provide hierarchic clustered heatmaps of T cell exhaustion **(A)**, TH1 signaling **(B)**, TH2 signaling **(C)**, and STAT3 pathways **(D)** with genes recognized in IPA to belong to these pathways.

The transcriptome data showed that T cell exhaustion signaling pathway was among the top pathways with a positive Z-score activated in the HIV exposed DCs from the DC-T cell cocultures (**Table 1**). The TH1 and TH2 Activation pathway and the Antigen presentation pathway were the top two canonical pathways affected in the DCs isolated from the HIV DC-T cell coculture but without a clear activation or inhibition pattern (**Supplementary Table 2**). When dissecting the individual donor profiles for DCs isolated from the mock and HIV treated DC-T cell cocultures, we found that there were increased Z-scores for IL12RB2, LAG3 and JAK2, factors that have been associated with T cell exhaustion (**Figure 8A**). The analysis also showed an increase in TH1 signaling (**Figure 8B**) and TH2 signaling (**Figure 8C**) with positive Z-scores for IL12RB2, KLRC1, JAK2 and STAT1. The STAT3 pathway was also affected (**Figure 8D**) with positive Z-scores for IL15RA, JAK2 and IL18R1.

### Transcriptomic profiles of HIV exposed mature DCs after DC-T cell interaction had a high match score with different data sets of type I IFNs polarized conditions

**Figure 9:**
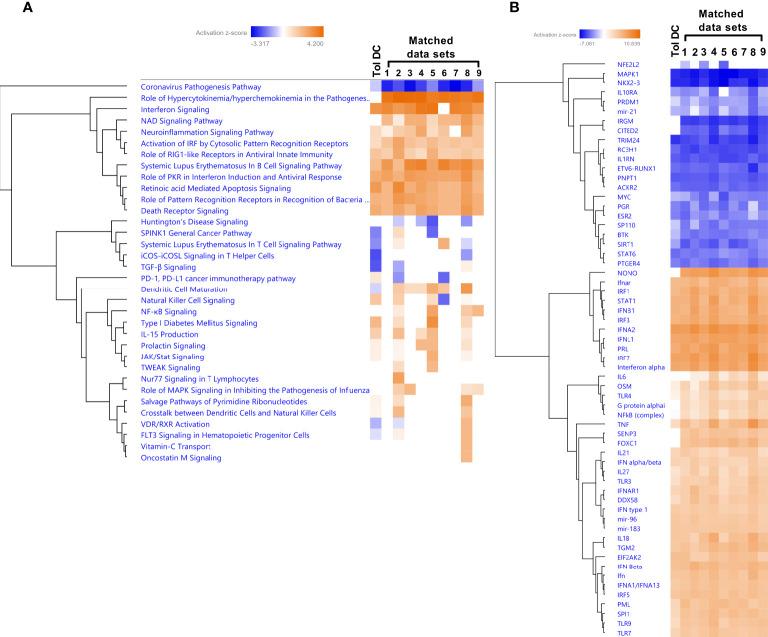
The HIV induced tolerogenic DCs had a high overlap with type I IFN exposure, and influenza infection data in a match analysis. Transcriptomic tolerogenic DC data set (Tol DC) generated from 8 individual donors and experiments was analyzed using a match analysis with at least 86% match in IPA and the matching data sets were (1) 6- normal control [lung] co-culture;influenza A;low glucose 4569, (2) 4- lung adenocarcinoma (LUAD) [alveoli] Infection_influenza A 16703, (3) 4- normal control [peripheral blood] culture medium 26822, (4) 10- disease control [peripheral blood] NA 4160, (5) 17- hepatocellular carcinoma (LIHC) [liver] IFN lambda 1 19882, (6) 3- normal control [lung] co-culture;high glucose;influenza A 4566, (7) 1- normal control [lung] co-culture; high glucose;influenza A 4564, (8) 1- pustular psoriasis [peripheral blood] NA 3612, 6- normal control [lung] co-culture;influenza A;low glucose 4569, and (9) 5- normal control [peripheral blood] NA 10198. Transcriptomic data set match analysis with top canonical pathways shown as hierarchic clustered heatmap with a threshold for p-values set to -log 1.3 (p<0.05) and Z-score 2 **(A)**. Transcriptomic data set match analysis with Upstream regulator shown as hierarchic clustered heatmap with a threshold for p-values set to log 1.3 (p<0.05) and Z-score 4 **(B)**.

We next performed match analysis to examine the match between our transcriptomic data of HIV exposed DCs from T cell cocultures with published data sets of human transcriptomic studies (**Figure 9A**). We set the cut off for the data sets with a minimum of 86% Canonical pathway Z-score match and minimum 76% Z-score overall score match. The top data sets included conditions characterized by type I IFN signaling; Influenza infection, IFN lambda treatment, and influenza A infection of Lung adenocarcinoma (**Supplementary Table 2**). Among the top five canonical pathways that were shared in the data sets were one inhibited and four activated. The Coronavirus pathogenesis pathway was inhibited, whereas Activation of IRF by Cytosolic Pattern Recognition Receptors, Role of RIG1-like Receptors in Antiviral Innate Immunity, Systemic Lupus Erythematosus in B cell signaling pathway, and Role of PKR in interferon Induction and Antiviral Response were activated. Role of hypercytokinemia/hyperchemokinemia in the pathogenesis of Influenza was enriched in the matched data sets but not in our data set (**Figure 9A**). The dendritic cell maturation pathway was inhibited in our data set but not in the matched data sets. Analysis of the Upstream regulators showed an activated Z-score for an array of type I IFN signaling molecules including IFNG, IFNA, IFNA2 and IRF7 for our and all matching data sets (**Figure 9B**). It was evident that type I IFN signaling was activated and regulators of inflammation including molecules such as NKX2-3, MAPK1, TRIM24, PNPT1, and IL1RN were suppressed in our and the matched data sets. IRGM and IRM1 were inhibited in the matched data sets but not in ours, whereas there was lower activation FOXC1, IL6, and NFkB (complex) in our data set, compared to the other data sets (**Figure 9B**).

## Discussion

DCs simultaneously express multiple activating and inhibitory factors, and depending on the balance, can activate or suppress the ensuing T cell responses (58). The resulting immune response is influenced by all the co-stimulatory and co-inhibitory receptors and ligands that are engaged and the subsequent network of signaling pathways that are activated. Our current investigations advance the understanding of the impaired immune activation that occurs when HIV is present during DC priming of T cells and the induction of suppressive T cells (49–51). We found that mere exposure of DCs to HIV had no significant effect on DC gene expression levels of the co-inhibitory factors, and is in accordance with previous findings, including our own, showing very little or almost no effect of HIV exposure alone on the DC phenotype (11, 59, 60). Instead, it was the contact with the suppressive T cells that induced the alteration of the mature DCs phenotype, expressing an array of co-inhibitory molecules including PDL1, HVEM, Gal-9, B7H3, PDL2, CD85, and CD30L. These results show that DC-T cell interactions induce a bidirectional expression of tolerogenic molecules. After the finding that DCs also adopted a suppressive phenotype in the system, we wanted to elucidate the mechanisms involved in the HIV induced upregulation of immune checkpoint molecules in DCs. In addition, we found that the DCs separated from the DC-T cell coculture after 24 crosstalk suppressed priming of naïve T cells and induced co-inhibitory molecules. To identify the affected signaling pathways responsible for the suppressive DC phenotype, either up- or downregulated, transcriptome analysis of DCs separated from HIV exposed or unexposed DC-T cell cocultures were performed. While it exists a wealth of information regarding the immunosuppressive markers on immune cells, there is a lack of understanding of the mechanisms that induce and direct immune suppression.

In our settings, T cell interaction induces strong and long-lasting type I IFN responses in the mature HIV exposed DCs, illustrated by the effect on a range of different IFN related genes in the transcriptomic data e.g., IFNG, IFNA, IFNA2 and IRF7. This sustained IFN response contrasts with what has been shown after HIV exposure of immature DCs (61), or in mature DCs after HIV exposure in the absence of T cells. Sustained type I IFN signaling could be one factor that triggers the shift to a tolerogenic DC phenotype with the enhanced expression of e.g., PDL1 (40, 62) and the subsequent induction of an immunosuppressive environment.

A large number of immunomodulatory molecules and pathways involved in controlling T cell response activation has been defined (63). Among the first co-stimulatory molecules defined to play an important role in T cell activation were CD80 and CD86, which both bind CD28 (64). Today, a lot is known about the effects that the engagement of co-inhibitory molecules such as PD1 and CTLA4 have on T cells. However, the reverse impact, i.e., the effect on antigen presenting cells (APCs) such as DCs, is less studied. The main and previously well-established immune checkpoint pathway molecule upregulated on antigen presenting cells during HIV infection is PDL1 (65). As shown here, PDL1 is not the only molecule of importance. However, PDL1 is among the most prominent negative immune checkpoint molecules expressed on the DC’s surface, and the PD1 axis is a potent suppressive pathway (64). The PD1-PDL1 interaction will send inhibitory signals into the T cells *via* PD1 and into the DCs *via* PDL1. Studies have shown that PDL1 can also interact with CD80 and contribute to T cell suppression (66, 67). PDL1 is upregulated in tumor cells, and immune cells such as APCs, including DCs, by e.g., IFNγ, IL-10, IFNα, and IFNβ (68–71). The role of PDL1 appears to be more complex than to suppress the T cell responses against tumor cells, since its presence can also protect against the immune suppressive effect of sustained type I and II IFN responses (72). The consequence of receptor-ligand interaction on the cell function is complex and context dependent. Surprisingly, engagement of the T cell co-stimulatory molecule CD28 can, under some circumstances, decrease T cell proliferation (73).

The increased expression of DcR2 on DCs present in HIV exposed cocultures is important since these decoy receptors reportedly bind to TRAIL and can facilitate T cell inhibition (74). We and others had previously shown that HIV infection could promote increased expression of TRAIL *in vitro* (49) and *in vivo* (75). Together, these results indicate a role of TRAIL-DcR2 signaling in HIV-associated immune suppression as seen herein from our *in vitro* observations. We found an elevated Gal-9 expression in the HIV exposed DCs after interaction with T cells in the coculture, and this β-galactosidase-binding lectin has been shown to promote an immune suppressive microenvironment with impaired T cell responses in different settings including HIV infection (76). Gal-9 is increased during acute HIV infection and the levels also remain elevated in individuals controlling the infection (77).

DCs orchestrate the fate of T cell responses and can efficiently regulate cellular effector functions through diverse mechanisms that range from synthesis of factors exerting broadly attenuating effects, to the induction of antigen-specific T cell responses that result in immune tolerance. The DCs are affected by the local microenvironment, which will also impact the type of T cell response induced. It is important to note that DC T cell interaction is a continuous process, and the same DC can activate multiple T cells (78, 79). So, the creation of suppressive DCs by the interaction with T cells in presence of HIV-1 in the lymphoid organs will influence the subsequent T cell responses activated, causing dysfunctional activation and impaired functionality of both HIV-1 specific and other antigen specific T cells (12). The DCs in our system, after only a few hours in cellular contact with T cells in the presence of HIV-1, create an environment causing impaired T cell responses. It is also known that the DCs will only survive for a few days in the lymph node influencing the T cell responses (79). We see the same in our *in vitro* system where most DCs are killed after a few days by the activated T cells.

The transcriptome profile of DCs from the HIV DC-T cell cocultures showed an inhibition of the TH2 pathway with decreased expression of multiple genes such as IL5, TNFRSF4 (OX40/CD134), CCR4, and CCR8 (29, 80). The TH1 pathway involving a variety of genes was less affected but still downregulated, suggesting a more general immune suppression not limited to TH2. We also found increased activation of T cell exhaustion pathways, in support of an HIV associated general immune suppression. The induction of T cell exhaustion is a problem in chronic HIV-infected individuals (81) and persists even in ART-treated individuals (82). In addition, there is a general immune suppression in many types of immune cells (3). Furthermore, there is an alteration in the T helper populations (83) and this might depend on the altered microenvironment at the site of DC activation.

The local microenvironment, characterized by multiple cellular interactions, bidirectional cell signaling and the release of inflammatory mediators, play a major role in the development of impaired immune function (84–86). There is a paucity in the understanding of the mechanisms leading to an immune suppressive microenvironment. It is clear from our study that the type I IFN-induced responses are stronger and last longer in HIV exposed DCs that have been in contact with T cells compared to the transient type I IFN activation seen when DCs are exposed to HIV without the cellular contact with T cells. It has been suggested that during chronic conditions such as viral persistence, the type I IFN can have suppressive effects on the immune responses (87). In addition, type I IFNs can induce tolerogenic/suppressive DCs in LCMV infection, tuberculosis, and cancer (40). Furthermore, the expression of PDL1 and the immune suppressive IL10, were highly dependent on type I IFN signaling in persistent viral infections (88). It is becoming clear from several studies, that for type I IFNs, the magnitude and timing of expression are essential for the outcome of the infection and quality of the immune responses (40, 87, 89). The antiviral type I IFN response and the inflammatory response are balanced by mutual crosstalk (90). The level of inflammation in the DCs and in our HIV coculture system is in general low and this could be the result of an imbalance reflected by a strengthened type I IFN responses in these systems. The role of type I IFNs in HIV pathogenesis has been debated. Early in HIV infection, increased IFNα levels are considered to restrict infection, i.e., they are beneficial, whereas elevated levels later in disease are associated with disease progression, with increased viral loads and decreased CD4^+^ T cell counts (91, 92). Furthermore, it has been suggested that there is a link between type I IFN and pathogenic disease and progression to AIDS in simian immunodeficiency virus (SIV) (93, 94). These findings in HIV infected individuals and SIV experimental models support a role of type I IFNs in the development of impaired immune responses and the induction of suppressive/tolerogenic DCs in our study.

DCs can also modulate T cell differentiation by modifying metabolic parameters in the T cell microenvironment by the production and release of retinoic acid (95). Our transcriptomic data showed activation of the retinoic acid mediated apoptosis signaling pathway. In addition, STAT3, STAT1, and STAT2 can be activated by type I IFNs and STAT3 is known to confer immunosuppressive functions (96). We identified an upregulation of STAT3 signaling in transcriptome profiles of the HIV exposed DCs after T cell interaction, and we know from our previous findings that STAT3 blockade in our *ex vivo* model of DC-T cell coculture can restore the DC T cell priming capacity after HIV exposure (50).

Our study has the limitations of being an *in vitro* model of the effects HIV exerts when DCs and naïve T cells interact, that aims to mimic the *in vivo* setting in the lymphoid organs. In addition, here we use monocyte derived DCs and not the mucosal tissue DCs or lymph node DCs (97), which should be the cells responsible for the priming of HIV specific immune response and spread of HIV to the T cells. The effect HIV exposed DCs exerted on the T cell responses was restricted to analysis of T cell proliferation. However, we have previously shown that reduced T cell proliferation after interaction with HIV exposed DCs is associated with a suppressive T cell phenotype (49–51).

**Figure 10:**
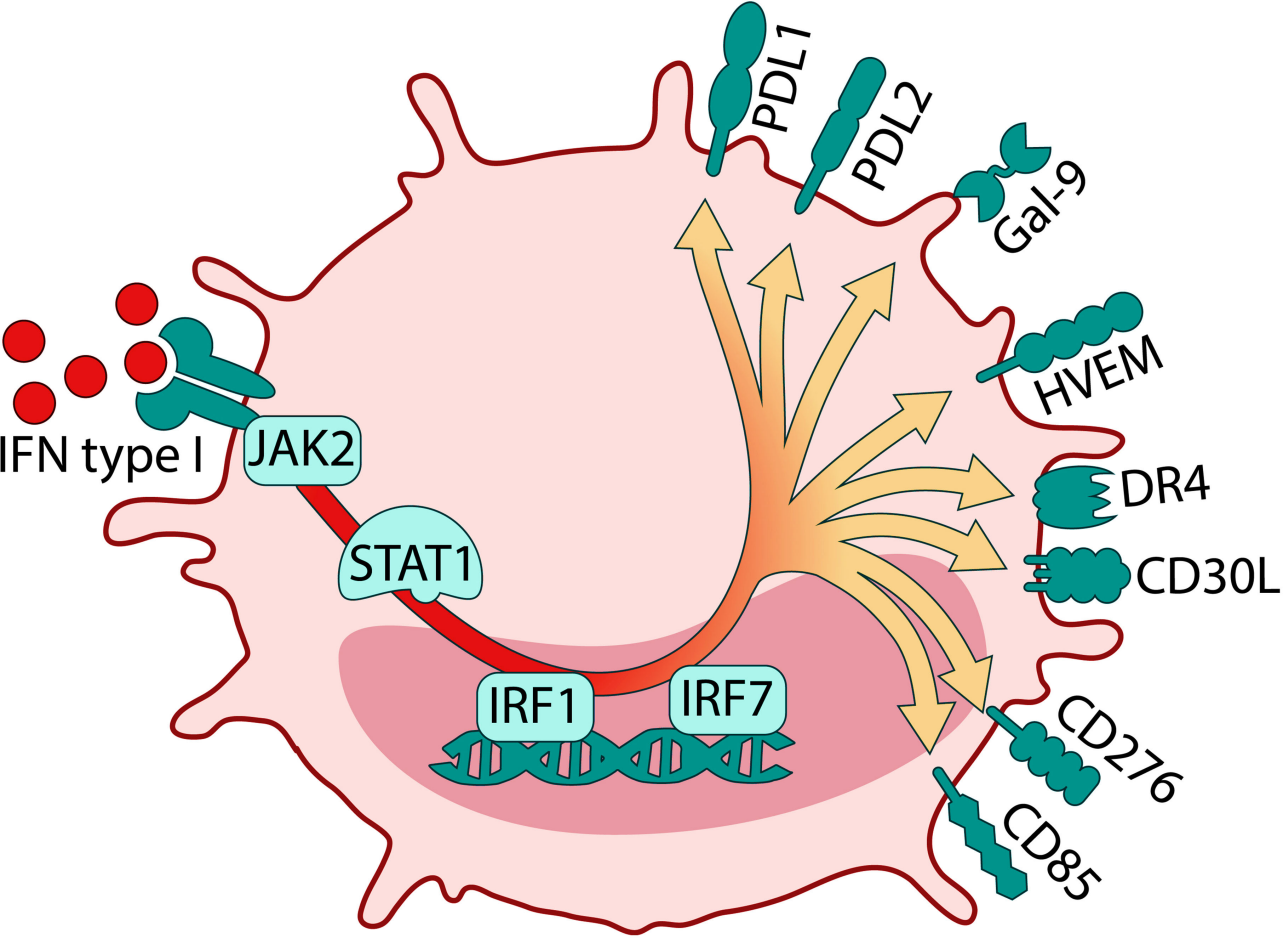
Graphical summary of HIV induced Tolerogenic DCs from DC T cell coculture. The crosstalk between DCs and T cells in the presence of HIV induces DCs with a tolerogenic phenotype and a strong type I IFN transcriptome profile.

Taken together, this study emphasizes that exposure of mature DCs to HIV significantly altered the overall balance in the expression of immune checkpoint molecules in favor of co-inhibitory molecules after the interaction with T cells. These findings shed a new light on the understanding of HIV that potentially skews DCs to differentiate into a tolerogenic/suppressive DC phenotype where cellular contact with the T cells alters their functionality and in which the IFN type I signaling could play an integral role (**see** **Figure 10** **for graphic summary**). Given the emergence of a wider network of co-inhibitory molecules in HIV infection, it is important now to investigate the specific role this environment plays in HIV pathogenesis in infected individuals and if targeting these pathways can reverse its negative effects.

## Data availability statement

The datasets presented in this study can be found in online repositories. The names of the repository/repositories and accession number(s) can be found in the article/**Supplementary Material**. The data presented in the study are deposited in the NCBI GEO repository, accession number GSE183774.

## Ethics statement

The studies involving human participants were reviewed and approved by Swedish Ethical Review Authority. Written informed consent for participation was not required for this study in accordance with the national legislation and the institutional requirements.

## Author contributions

CS, SN, MG, PB, KC, ES, RE, and ML performed experiment and/or analyzed data. CS, SN, MG, KC, ES, and ML wrote and edited the manuscript. All authors contributed to the article and approved the submitted version.

## Funding

This work has been supported by grants through: ML: AI52731, The Swedish Research Council, Physicians against AIDS Research Foundation, The Swedish International Development Cooperation Agency; SIDA SARC, VINNMER for Vinnova, Linköping University Hospital Research Fund, ALF, the Swedish Society of Medicine, and SN: Molecular Infection Medicine Sweden.

## Acknowledgments

We thank the Biological Products Core of the AIDS and Cancer Virus Program, SAIC Frederick, Inc., National Cancer Institute, Frederick, MD, USA for generously providing HIV. The data handling was enabled by resources provided by the Swedish National Infrastructure for Computing (SNIC) at UPPMAX partially funded by the Swedish Research Council through grant agreement no. 2018-05973.

## Conflict of interest

The authors declare that the research was conducted in the absence of any commercial or financial relationships that could be construed as a potential conflict of interest.

## Publisher’s note

All claims expressed in this article are solely those of the authors and do not necessarily represent those of their affiliated organizations, or those of the publisher, the editors and the reviewers. Any product that may be evaluated in this article, or claim that may be made by its manufacturer, is not guaranteed or endorsed by the publisher.
