# Supplementary material for: HIV-1 induction of tolerogenic dendritic cells is mediated by cellular interaction with suppressive T cells
Source: Front Immunol. 2022 Aug 10;13:790276. doi: 10.3389/fimmu.2022.790276 (PMC9399885; doi:10.3389/fimmu.2022.790276)
Supplement: Supplementary file 1 [file DataSheet_1.docx]

**Supplementary figures Svanberg et al**

**
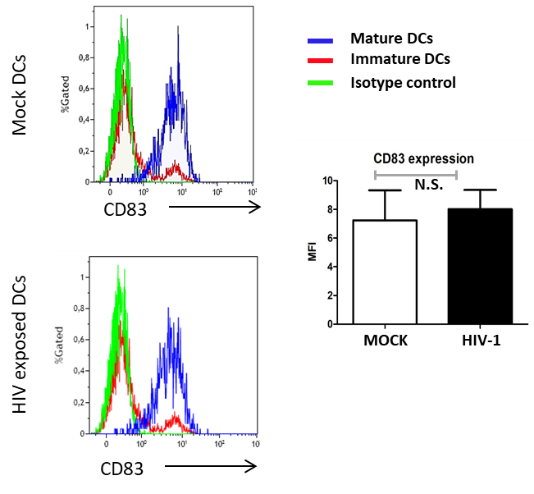
**

**Supplementary Figure 1.**

**HIV-1 does not alter the DCs CD83 expression/maturation status compared to mock.**

Immature and mature DCs were exposed to HIV for 24h and stained with anti-CD83 antibody or isotype control antibody and analysed by flow cytometry and visualized by representative histograms (left) and MFI graph (right). N=3.

**Supplementary Figure 2.** **Upregulation of negative costimulatory molecule and modulatory factors occurs fast in DCs after their interaction with the day 7 HIV coculture.**

Expression of PDL-1 and IDO mRNA levels after 5h DC exposure to day 7 co-cultures were investigated with qPCR. Expression is shown as relative expression compared with its respective unexposed (mock) control which are stated as one. Results are shown as mean with standard deviation N=3.

**
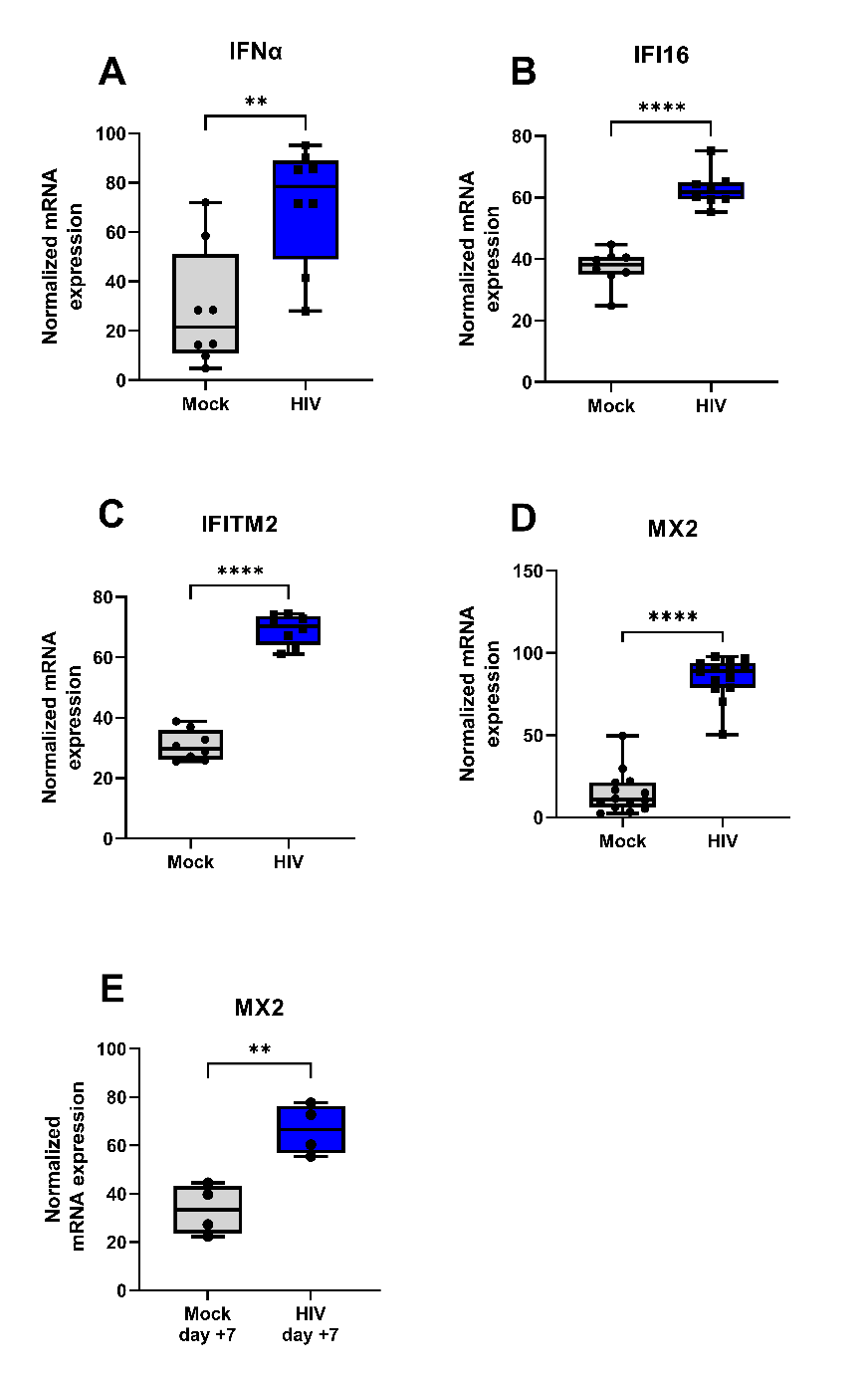
**

**Supplementary Figure 3.**

**Type I IFN and IFN stimulated genes are upregulated in the HIV exposed DC-T cell coculture.**

The mRNA gene expression levels of IFNα (**A**), IFITM2 (**B**), IFI16 (**C**), and MXA2 (**D**) were examined by qPCR in mock and HIV-1 exposed DC-T cell cocultures at day 8 (N=8-14). MX2 gene expression levels were investigated at day 7 in cocultures with naïve T cells stimulated with DCs purified from day 8 mock and HIV DC-T cell cocultures (N=4) (**E**). The qPCR data was normalized as ratios of 100%. Shown are min to max and all data points. **P<0.005, ****P<0.0001, unpaired t-test.

**Supplementary Table 1. Genes and primer sequences**

| Primer (gene name) | Sequence |
| --- | --- |
| *gag* (HIV-1 gag) forward | GGC TTT CAG CCC AGA AGT AAT ACC C |
| *gag* (HIV-1 gag) reverse | TTG CAT GGC TGC TTG ATG TCC CC |
| *CD274* (PDL1) forward | GCC CGT GGG ATG CAG GCA AT |
| *CD274* (PDL1) reverse | CAC AGG CGT CGA TGA GCC CC |
| *TNFRSF14* (HVEM) forward | CCG ACG TCT TGA GGC TGG TGC |
| *TNFRSF14* (HVEM) reverse | TGC CTG GAG GGC AGG GTT CA |
| *LGALS9* (Galactin-9) forward | GAT GAG AAT GCT GTG GTC CG |
| *LGALS9* (Galactin-9) reverse | GAA GCC GCC TAT GTC TGC A |
| *TNFRSF10D* (Dcr-2) forward | CGC TCG AGC AGG GCG CTA TC |
| *TNFRSF10D* (Dcr-2) reverse | AGA GTC AAC CCG GAC CGG CA |
| *TNFRSF10A (*Dr4) forward | TCC CTG CAC CAC GAC CAG GA |
| *TNFRSF10A* (Dr4) reverse | CCT CTG GGG CAC CCT CTG CT |
| *PTGS2* (COX-2) forward | CTT CAA AAT AAG CTT GAA TTC AGG |
| *PTGS2* (COX-2) reverse | CTT TTT GAT AAT TTA ATA ATT TCA ATC TTC TGT TTC |
| *IDO1* (IDO) forward | GCA AAT GCA AGA ACG GGA CAC T |
| *IDO1* (IDO) reverse | TCA GGG AGA CCA GAG CTT TCA CAC |
| *TDO2* (TDO) forward | ACT CCC CGT AGA AGG CAG CGA |
| *TDO2* (TDO) reverse | TCT TTC CAG CCA TGC CTC CAC T |
| *MX2* (MX2) forward | CAG AGG CAG CGG AAT CGT AA |
| *MX2* (MX2) reverse | TGA AGC TCT AGC TCG GTG TTC |
| *IFITM2* (IFITM2) forward | ATG AAC CAC ATT GTG CAA ACC T |
| *IFITM2* (IFITM2) reverse | CGG AGT AGG CGA ATG CTA TGA A |
| *IFNα* (IFNα) forward | ACC TAG AGG CCG AAG TTC AAG GT |
| *IFNα* (IFNα) reverse | GCA CCA GCA CGG CCA TCA G |
| *IFI16* (IFI16) forward | GCC GGC ATG TCC ACA GCC AT |
| *IFI16* (IFI16) reverse | TGG CCA CTG TTT TCG GGT TCT CA |
| *ACTB* (Actin) forward | AGA GGG AAA TCG TGC GTC AC |
| *ACTB* (Actin) reverse | CAA TAG TGA TGA CCT GGC CGT |
| *GAPDH* (GAPDH) forward | CCA CCA TGG AGA AGG CTG GGG CTC |
| *GAPDH* (GAPDH) reverse | AGT GAT GGC ATG GAC TGT GGT CAT |

**Supplementary Table 2.** **Top Canonical pathways**

**Supplementary Table 3.** **Matched analysis with published data sets with high** **overlap in Z-Score**

Selected on Human disease data base.

CP = canonical pathway Z-Score, UR = upstream regulators Z-Score
